# Factors Associated with False Negative Results in Serum Pepsinogen Testing for Precancerous Gastric Lesions in a European Population in the GISTAR Study

**DOI:** 10.3390/diagnostics12051166

**Published:** 2022-05-07

**Authors:** Danute Razuka-Ebela, Inese Polaka, Ilva Daugule, Sergei Parshutin, Daiga Santare, Inguna Ebela, Dace Rudzite, Reinis Vangravs, Rolando Herrero, Jin Young Park, Marcis Leja

**Affiliations:** 1 Faculty of Medicine, University of Latvia, Jelgavas iela 3, LV-1004 Riga, Latvia; ilva.daugule@lu.lv (I.D.); daiga.santare@lu.lv (D.S.); inguna.ebela@gmail.com (I.E.); marcis.leja@lu.lv (M.L.); 2 Institute of Clinical and Preventive Medicine, University of Latvia, Jelgavas iela 3, LV-1004 Riga, Latvia; inese.polaka@lu.lv (I.P.); sergejs.parsutins@lu.lv (S.P.); dacerudzite2008@inbox.lv (D.R.); reinis.vangravs@lu.lv (R.V.); 3 Riga East University Hospital, Hipokrāta iela 2, LV-1038 Riga, Latvia; 4 International Agency for Research on Cancer, 150 Cours Albert Thomas, CEDEX 08, 69372 Lyon, France; rherrero@acibcr.com (R.H.); parkjy@iarc.fr (J.Y.P.); 5 Agencia Costarricense de Investigaciones Biomedicas, Fundación INCIENSA, Avenida 9a Calles 64-68, San Jose 2250, Costa Rica

**Keywords:** pepsinogen, precancerous gastric lesions, *Helicobacter pylori*, smoking, sensitivity, GISTAR, Latvia

## Abstract

The accuracy of plasma pepsinogen (Pg) as a marker for precancerous gastric lesions (PGL) has shown variable results. We aimed to identify factors associated with false negative (FN) cases in Pg testing and to adjust cut-off values for these factors in order to improve Pg yield. Plasma Pg was measured and upper endoscopy with biopsy was performed within the “Multicentric randomized study of *Helicobacter pylori* eradication and pepsinogen testing for prevention of gastric cancer mortality: the GISTAR study”. A multivariable logistic model was built for FN and multiple factors. Values of Pg were compared and sensitivity and specificity were calculated using pre-existing Pg cut-offs for factors showing strong associations with FN. New cut-offs were calculated for factors that showed substantially lower sensitivity. Of 1210 participants, 364 (30.1%) had histologically confirmed PGL, of which 160 (44.0%) were FN. Current smokers, men, and *H. pylori* positives were more likely FN. Smoking in *H. pylori* negatives was associated with a higher Pg I/II ratio and substantially lower sensitivity of Pg testing than in other groups. Adjusting Pg cut-offs for current smokers by *H. pylori* presence improved sensitivity for detecting PGL in this group. Our study suggests that adjusting Pg cut-offs for current smokers by *H. pylori* status could improve Pg test performance.

## 1. Introduction

Although the global incidence of gastric cancer has been gradually decreasing, gastric cancer is the fourth leading cause of cancer related death [1]. In Europe, less than 20% of cases are diagnosed early [2]. Even in high income countries, gastric cancer survival is low, showing that emphasis must be placed on preventive strategies. The detection of precancerous gastric lesions is a secondary preventive strategy. As nationwide endoscopic screening programmes are not expected to be cost-effective in European countries, non-invasive pepsinogen (Pg) testing is a promising option [1,2].

Serum pepsinogen (Pg) is currently the best non-invasive method of assessing gastric mucosal status [3]. Low Pg I and Pg I/II ratio are suggestive of atrophic gastritis and have been proposed for identifying individuals at increased risk of gastric cancer [4,5].

However, the sensitivity and specificity of serum Pg as a marker for precancerous gastric lesions and gastric cancer vary, with significant heterogeneity between studies. Two meta-analyses reported that pepsinogens exhibit a moderate diagnostic yield, possibly due to both methodological and population based differences [6,7]. Previous analysis within the GISTAR study in Latvia on the diagnostic performance of Pg testing in identifying moderate to severe gastric atrophy showed a sensitivity and specificity of 52% and 90% (AUC 0.77), with adjustments made to cut-off values not showing any significant improvement [8].

Most studies on factors potentially affecting Pg values have been conducted in Asian populations. Pepsinogen levels can increase with higher levels of inflammation [9]. Chronic smoking increases gastric secretion and can increase pepsinogen, especially Pg I [10,11]. Several studies in Asia report higher Pg I and Pg II levels in those *H. pylori* positive [12,13,14,15]. *H. pylori* infection is associated with atrophic gastritis and is the main risk factor in the development of gastric cancer [16,17].

Higher Pg I and Pg II levels have been reported in men compared to women [12,14,15,18,19]. In some studies, Pg levels gradually increased with age [14,15], while Pg I/II ratio decreased [12,15]. In a study in China, Tong et al. (2021) reported regional differences in Pg levels, suggesting that this could be associated with ethnicity and different dietary habits [20].

It seems that only Tong et al. (2017) has reported improved Pg accuracy in detecting atrophic gastritis by establishing different pepsinogen cut-off values for those *H. pylori* positive and negative in China [9]. There seem to be no studies that have tried to adjust pepsinogen cut-off values based on more than just *H. pylori* status.

This study aims to identify factors associated with false negative cases of Pg testing and to adjust Pg cut-off levels in order to improve the detection of precancerous gastric lesions.

## 2. Methods

### 2.1. Study Design

Participants aged 40 to 64 years that underwent plasma pepsinogen testing and upper endoscopy within the main “Multicentric randomized study of *H. pylori* eradication and pepsinogen testing for prevention of gastric cancer mortality: the GISTAR study” in Latvia from 2016 to 2021 were included in the analysis. Participants were excluded if they had undergone *H. pylori* eradication within the past year, had a history of gastric resection or gastric cancer, or signs of serious disease. More detailed information on the GISTAR study can be found elsewhere [21].

Self-reported data covering socio-economic characteristics, lifestyle, and medical history was obtained with a questionnaire. The choice of factors included in the analysis was based on both a review of the literature and our previous findings [22]. Age, gender, level of education, income, and employment were included in the analysis. Participants were grouped into current, former, and never smokers. Alcohol was included in the analysis as there are reports of an association with Pg levels [23]. Participants were asked to recall how often, how much, and what type of alcohol they had consumed in the past year. The amount of each type in milliliters was converted and combined into total grams of ethanol per week, modeled at an increase of 10 g.

A large selection of dietary habits was included: having at least 400 g of fruit and vegetables and at least 200 g of dairy daily during the previous week, very hot food or drinks and spicy food (times per week), number of meals per day, and the frequency of having several products daily during an average week in the previous year assessed with a food frequency questionnaire (never, once, twice a month, one, two to four, five to six times a week, daily) which included kefir, red meat, poultry, pickled, cured, salted and smoked products, onion and spring onion, garlic, sweetened beverages, coffee, and tea. BMI was included because of reports of differences in Pg values between categories [15,24]. The height and weight of participants were measured on site. Self-reported history of proton pump inhibitor (PPI) use in the previous month was included because of the effect of PPIs on gastric secretion [25].

Participants were tested for Pg I and Pg II by latex-agglutination test-system (Eiken Chemical, Tokyo, Japan) and Pg I/Pg II ratio was calculated. Based on previously established Pg cut-off values, participants with Pg I/Pg II ≤ 2 and Pg I ≤ 30 ng/mL were considered at increased risk of precancerous gastric lesions and offered upper endoscopy with biopsy as part of the GISTAR study [19]. Gastric biopsy samples were evaluated by two independent pathologists according to the updated Sydney system and OLGA (Operative Link for Gastritis) and OLGIM (Operative Link for Intestinal Metaplasia) risk stratification staging systems [26,27]. For quality assurance, a third expert pathologist participated in the evaluation of random slides and in cases with advanced lesions. The presence of *H. pylori* was determined by modified Giemsa staining. In addition to routine methods, samples with intestinal metaplasia were stained with high iron diamine-alcian blue and classified as type I (complete), type II (incomplete), and type III (incomplete) [28].

In accordance with the guidelines for the “Management of epithelial precancerous conditions and lesions in the stomach (MAPS II)” that provide recommendations for surveillance strategies, participants with high-risk stages (OLGA/OLGIM III-IV), dysplasia, as well as participants with extensive atrophy or intestinal metaplasia and incomplete metaplasia were placed in the precancerous gastric lesions group [29].

For the current study, participants were divided into two groups. Participants with decreased Pg levels (defined as Pg I/Pg II ≤ 2 and Pg I ≤ 30 ng/mL) and precancerous gastric lesions confirmed by biopsy were placed in the true positive group (TP), while participants with unchanged Pg levels and precancerous gastric lesions were placed in the false negative group (FN).

### 2.2. Statistical Analysis

For qualitative variables, the percent of participants in each group (FN and TP) is presented. For quantitative variables, means and standard deviations (SD) or medians and interquartile range (IQR) were calculated depending on the distribution. Pearson’s chi-square, Mann–Whitney, and Kruskal–Wallis tests were used to identify differences between FN and TP. Factors significantly associated with FN in univariate analysis were included in a multivariable regression model, adjusted for age, and odds ratios (OR) were calculated. Significance at α < 0.10 level was considered statistically significant in univariate analysis and at α < 0.05 level for multivariate analysis.

Median Pg I, Pg II, and Pg I/II values were compared by *H. pylori* presence and smoking status. Based on histology participants were divided into those with and those without precancerous gastric lesions. In each group, median Pg I and Pg I/II values were compared between those *H. pylori* positive and *H. pylori* negative by smoking status. Analysis was performed using SPSS version 21.0 [30].

### 2.3. Analysis of Pg Test Sensitivity and Specificity

Participants were divided into groups based on *H. pylori* and smoking status. Using the pre-existing pepsinogen cut-offs (Pg I/Pg II ≤ 2 and Pg I ≤ 30 ng/mL) receiver operating characteristic (ROC) curves were calculated using the results of regression curves for Pg I/II ratio and Pg I and evaluated by calculating the area under the curve (AUC). Supplementary analysis was performed stratifying by sex and age.

For the groups that had a sensitivity or specificity under 65%, Pg cut-offs were adjusted using Youden’s index to find Pg I and Pg I/II cut-offs that provide the highest sensitivity and specificity in identifying precancerous gastric lesions.

## 3. Results

Of a total of 1210 participants, 364 (30.1%) had precancerous gastric lesions confirmed by biopsy. Of these 160 (44.0%) were FN, while 204 (56.0%) were TP. Of all the participants, 61 (5.0%) were false positive.

When compared to the TP group, participants in the FN group were more likely to be men, current smokers, and *H. pylori* positive (Table 1). Participants in the FN group consumed more alcohol, less fruit and vegetables daily, and had lower earnings than TP.

**Figure 1:**
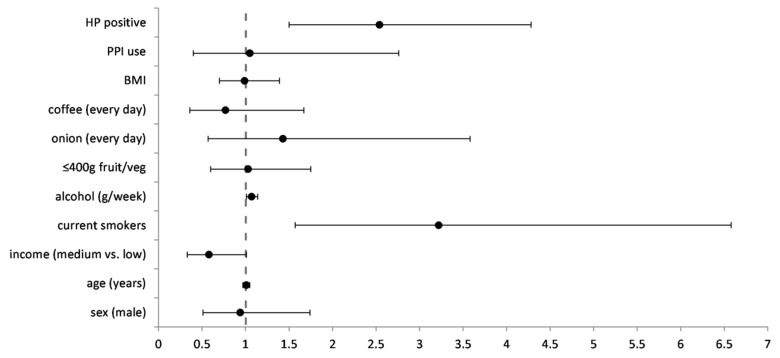
Multivariable regression model for false negative cases in detecting precancerous gastric lesions with pepsinogen testing (*n* = 293). FN was compared against TP. OR—odds ratio, adjusted for all the factors in the model, CI—confidence interval, HP—*Helicobacter pylori*, PPI—proton pump inhibitor, BMI—body mass index.

Factors significantly associated with FN were included in multivariate analysis and adjusted for age (Figure 1, Supplementary Table S1). In multivariate analysis, FN were significantly associated with current smoking, alcohol consumption and the presence of *H. pylori*. Although there were more men in the FN group and men were more likely to be current smokers, no significant differences were found between FN and TP when further stratifying participants by sex.

### 3.1. Differences in Pepsinogen Values by H. pylori and Smoking Status

Median Pg I and Pg II values were higher, while Pg I/II ratio was lower in *H. pylori* positive participants than *H. pylori* negative participants (Table 2). Smokers had higher median Pg I and Pg II values, as well as a higher median Pg I/II ratio than never smokers.

In order to further investigate differences in Pg values associated with both *H. pylori* and smoking, Pg I values and Pg I/II ratios were compared after dividing participants into two groups—with and without precancerous gastric lesions (Table 3 and Supplementary Table S2).

Median Pg I values were higher in *H. pylori* positive than negative participants in both groups—with and without precancerous gastric lesions.

In the group without precancerous gastric lesions, *H. pylori* positive participants had lower Pg I/II ratio values than *H. pylori* negative participants regardless of smoking status. In the group with precancerous gastric lesions, the median Pg I/II ratio was lower for *H. pylori* positives than negatives only for current smokers. The opposite was observed for former and never smokers—the Pg I/II ratio was higher for those *H. pylori* positive than those negative.

### 3.2. Sensitivity and Specificity for Detecting Precancerous Gastric Lesions by H. pylori and Smoking Status

When comparing the sensitivity and specificity of pre-existing Pg cut-off values (Pg I/Pg II ≤ 2 and Pg I ≤ 30 ng/mL) for the detection of precancerous gastric lesions by *H. pylori* presence and smoking status, substantial differences were observed. The sensitivity of the Pg test for detecting precancerous lesions was substantially lower for current smokers in comparison to other groups and was affected by *H. pylori* presence (Table 4).

The sensitivity of the Pg test was consistently lower for current smokers when using the pre-existing cut-offs. New cut-offs were calculated using Youden’s index to find Pg I and Pg I/II cut-offs that provide the highest sensitivity and specificity in identifying precancerous gastric lesions (Table 5 and Table 6). New cut-offs were also calculated for the groups without substantially decreased Pg test sensitivity (above 65%) for the purpose of comparison.

## 4. Discussion

When using pre-existing Pg cut-offs to identify precancerous gastric lesions in the study population, there was a substantial number of FN cases (160/367 or 43.6%), leaving significant room for the improvement of Pg diagnostic accuracy. Factors that remained significantly associated with FN were *H. pylori*, current smoking, and alcohol consumption.

The observation that Pg I and Pg II levels are higher in *H. pylori* positives than negatives coincides with reports in several other studies [12,14,15]. A larger increase in Pg II than Pg I in *H. pylori* positive compared to *H. pylori* negative participants could explain the lower Pg I/II ratio in *H. pylori* positives (110% versus 42% increase, respectively, Table 2). This is also supported by the idea that inflammation associated with *H. pylori* can increase Pg II secretion, as the gastric antrum is both the location of Pg II secreting cells and provides the best conditions for *H. pylori* survival [14]. A recent study in China by Yu et al. (2021) reported a twofold increase in Pg II compared to Pg I when comparing *H. pylori* negatives to positives [15].

In our study, smoking was associated with an increase in Pg I and Pg II, as well as Pg I/II ratio. A higher Pg I/II ratio in current smokers could be explained by a proportionally larger difference in Pg I values than Pg II values when comparing never smokers to current smokers (25% vs. 17.5% increase, respectively, Table 2). Smoking can increase gastric secretion and has been associated with an increase in Pg I in particular [8,9].

Upon further analysis, we observed that *H. pylori* and smoking not only displayed independent effects on Pg levels, but *H. pylori* seemed to modify the effect current smoking had on the sensitivity of Pg testing. This suggests that it might be insufficient to consider *H. pylori* and smoking separately when investigating FN in Pg testing.

In our study, median Pg values in the group without precancerous gastric lesions followed a similar pattern to that of the general study population—the median Pg I value was higher, and Pg I/II was lower in *H. pylori* positives than negatives, regardless of smoking status (Table 3 and Table S2), which could be explained by higher Pg II values in those *H. pylori* positive, as discussed previously.

In the precancerous gastric lesion group, the pattern of lower Pg I/II ratio in *H. pyl*ori positives than negatives was only observed for current smokers, with the opposite in former and never smokers (higher Pg I/II ratio in *H. pylori* positives). *H. pylori* negative current smokers had a substantially higher Pg I/II value than *H. pylori* negative never smokers. Pg I values were higher in the current smokers’ group not only in the general population, but also substantially higher among current smokers than never smokers for participants with precancerous gastric lesions. In the precancerous gastric lesion group, the higher median Pg I/II ratio in *H. pylori* negative current smokers could possibly be explained by a smoking-mediated increase in Pg I that is not offset by a *H. pylori*-mediated increase in Pg II, therefore maintaining a higher Pg I/II ratio on average.

This explanation seems to be supported by the analysis of Pg test sensitivity. For *H. pylori* negative current smokers, Pg testing showed substantially lower sensitivity for the pre-existing Pg I/II and Pg I cut-offs, possibly due to the higher Pg I levels associated with smoking. For *H. pylori* positive smokers, test sensitivity was still substantially lower for Pg I, but less so for Pg I/II, possibly due to the relatively higher Pg II levels associated with *H. pylori*. The comparatively higher Pg I levels in current smokers could be used to explain why smokers are more likely to be false negative than never smokers.

Our findings suggest that determining *H. pylori* status in current smokers and using adjusted Pg cut-offs for this subpopulation may improve the sensitivity of the Pg test in detecting precancerous gastric lesions. The number and variety of factors included as well as histological confirmation of *H. pylori* are strengths of the study. However, despite the sample size, it is still necessary to repeat the study with a larger population to assess whether the adjustments made to Pg cut-off values will provide a similar improvement in detecting precancerous gastric lesions and could be applicable to different populations. In our study, the most significant improvements in Pg test sensitivity were made with the new Pg I cut-offs, but at the expense of specificity (Table 6). The improvements in sensitivity for Pg I/II ratio were comparatively smaller and were more pronounced in *H. pylori* negative current smokers. Other individual factors may be affecting Pg test performance. We did not find significant differences in Pg values for FN and in sensitivity analyses for the detection of precancerous gastric lesions when stratifying by age and sex, although some studies have reported associations [14,15,31]. Analysis of sensitivity and specificity by smoking and *H. pylori* status with further stratification by sex resulted in groups that were too small for analysis.

The study was cross-sectional in nature and so conclusions cannot be made regarding cause and effect. Self-reported variables such as a history of PPI use, consumption of alcohol and different foods may not be reported accurately due to recall bias and error. The average reported values for alcohol consumption were below averages in national surveys, suggesting that participants may have underreported consumption.

## 5. Conclusions

In our study, both median Pg levels and the sensitivity of the Pg test in detecting precancerous gastric lesions were affected by current smoking and the presence of *H. pylori* infection. Adjusting Pg cut-off values for current smokers by *H. pylori* status improved the sensitivity of the Pg test. This suggests that adjusting Pg cut-offs for smokers could be used to improve Pg performance in detecting precancerous gastric lesions. Repeating the study in other populations is necessary to assess whether this approach can be used in other countries.

## Figures and Tables

**Table 1:** Comparison of factors between false negative and true positive cases in detecting precancerous gastric lesions by pepsinogen testing.

Variables, *n* (%)	Population, *n* = 1210	True Positives (TP), *n* = 204	False Negatives (FN), *n* = 160	*p*-Value *
Sex (male)	168 (46.8)	79 (38.7)	91 (56.9)	<0.01
Age in years (median, IQR)	52.0 IQR 11	56.0 IQR 10	54.5 IQR 10	0.16 **
Income (Euros) ^a^				<0.01
<250	65 (45.1)	54 (28.9)	65 (45.1)	
250–500	66 (45.8)	98 (52.4)	66 (45.8)	
500–1000	13 (9.0)	31 (16.6)	13 (9.0)	
>1000	0 (0.0)	4 (2.1)	0 (0.0)	
Unemployed	47 (13.1)	26 (12.7)	21 (13.1)	0.98
Employed	272 (75.8)	156 (76.5)	121 (75.6)	
Retired	40 (11.1)	22 (10.8)	18 (11.2)	
Never smokers	208 (57.9)	136 (66.7)	76 (47.5)	<0.01
Former smokers	71 (19.8)	42 (20.6)	29 (18.1)	
Current smokers	80 (22.3)	26 (12.7)	55 (34.4)	
Alcohol (g/week)	11.9 IQR 31.4	10.1 IQR 24.2	13.5 IQR 45.4	0.05 **
At least 400 g fruit and vegetables daily	194 (54.0)	120 (58.8)	78 (48.8)	0.06
At least 200 g dairy products daily	208 (57.9)	124 (60.8)	88 (55.0)	0.27
Onion and spring onion				
once a week	40 (12.2)	22 (12.7)	18 (11.4)	0.04
2–4 times per week	135 (41.3)	62 (35.8)	76 (48.1)	
5–6 times per week	94 (28.7)	60 (34.7)	34 (21.5)	
every day	58 (17.7)	29 (16.8)	30 (19.0)	
Coffee				
once a week	49 (13.7)	20 (9.8)	29 (18.2)	0.06
2–6 times per week	64 (17.9)	36 (17.6)	29 (18.2)	
every day	245 (68.4)	148 (72.5)	101 (63.5)	
BMI (kg/m^2^)				
<18.5 (underweight)	0	0	0	0.06
18.5–24.9 (normal)	88 (24.9)	50 (24.6)	38 (24.4)	
25.0–29.9 (overweight)	132 (37.3)	67 (33.0)	69 (44.2)	
≥30 (obese)	134 (37.9)	86 (42.4)	49 (31.4)	
PPI in previous month	26 (7.3)	12 (5.9)	15 (9.4)	0.20
*H. pylori* positive (biopsy)	205 (57.1)	95 (46.6)	113 (70.6)	<0.01

* Differences obtained with χ^2^ test comparing the false negative group (FN) to the true positive group (TP). ** Differences obtained using Mann–Whitney U test. ^a^ monthly household income per household member after tax. IQR—interquartile range, BMI—body mass index, PPI—proton pump inhibitor.

**Table 2:** Comparison of pepsinogen values by *H. pylori* presence and smoking status in the general study population.

	Pg I (ng/mL) Median, IQR	Pg II (ng/mL) Median, IQR	Pg I/II Ratio Median, IQR
***H. pylori***			
Positive	47.3, 31.4	18.7, 11.7	2.6, 1.5
Negative	33.3, 25.6	8.9, 5.1	4.0, 3.3
*p*-value *	<0.01	<0.01	<0.01
**Smoking**			
never smokers	36.8, 30.8	12.6, 12.4	2.8, 2.5
former smokers	41.5, 31.1	13.2, 11.4	2.9, 2.4
current smokers	46.0, 28.0	14.8, 12.5	3.3, 2.2
*p*-value **	<0.01	<0.01	0.01

* Differences obtained using Mann–Whitney U test to compare groups. ** Differences obtained using Kruskal–Wallis test to compare groups. Pg—pepsinogen, IQR—interquartile range.

**Table 3:** Comparison of median Pg I/II ratio by smoking status and *H. pylori* presence for participants with and without precancerous gastric lesions.

	No Precancerous Gastric Lesion PgI/II ng/mL Median, IQR			Precancerous Gastric Lesion PgI/II ng/mL Median, IQR		
	*H. pylori* Positive	*H. pylori* Negative	*p* Value *	*H. pylori* Positive	*H. pylori* Negative	*p* Value *
study population	2.9 IQR 1.4	4.4 IQR 1.8	<0.01	1.7 IQR 1.6	0.9 IQR 1.8	0.01
never smokers	2.8 IQR 1.4	4.3 IQR 1.8	<0.01	1.5 IQR 1.5	0.8 IQR 0.9	<0.01
former smokers	2.9 IQR 0.9	4.7 IQR 1.4	<0.01	1.8 IQR 1.8	0.8 IQR 0.8	0.02
current smokers	3.1 IQR 1.5	4.7 IQR 2.0	<0.01	1.9 IQR 1.5	3.4 IQR 4.1	0.02

* Differences obtained using Mann–Whitney U test to compare *H. pylori* positive and negative groups (histology). IQR—interquartile range.

**Table 4:** Sensitivity and specificity for detecting precancerous gastric lesions by smoking status and *H. pylori* presence when using pre-existing pepsinogen cut-off values.

Sensitivity (%, 95% CI), Specificity (%, 95% CI); Area under ROC Curve (AUC)		
	PgI/II ≤ 2	PgI ≤ 30 ng/mL
All participants	65.4 (60.3–70.3), 87.1 (84.7–89.3); 0.82	62.6 (57.4–67.6), 81.6 (78.8–84.1); 0.75
Never smokers, *H. pylori* positive	65.8 (56.3–74.4), 80.1 (74.8–84.7); 0.80	57.0 (47.4–66.3), 85.34 (80.51–89.36); 0.76
Never smokers, *H. pylori* negative	81.6 (72.5–88.7), 92.4 (88.2–95.4); 0.88	90.8 (83.3–95.7), 69.9 (63.6–75.7); 0.89
Former smokers, *H. pylori* positive	56.8 (41.0–71.7); 83.5 (74.6–90.3); 0.79	50.0 (34.6–65.4), 87.6 (79.4–93.4); 0.74
Former smokers, *H. pylori* negative	81.5 (61.9–93.7), 94.9 (87.5–98.6); 0.96	88.9 (70.8–97.7), 78.5 (67.8–86.9); 0.93
Current smokers, *H. pylori* positive	52.0 (37.4–66.3), 87.0 (79.2–92.7); 0.76	32.00 (19.52–46.70), 91.7 (84.8–96.1); 0.63
Current smokers, *H. pylori* negative	32.3 (16.7–51.4), 93.3 (83.8–98.2); 0.68	38.7 (21.9–57.8), 86.7 (75.4–94.1); 0.68

CI—confidence interval, ROC—receiver operating characteristic curve, Pg—pepsinogen.

**Table 5:** Pepsinogen cut-off values adjusted for detecting precancerous gastric lesions in smokers by *H. pylori* presence.

New Pg Cut-Off; Sensitivity (%), Specificity (%); Youden’s Index		
	Pg I/Pg II	Pg I (ng/mL)
Never smokers, *H. pylori* positive	≤1.76; 60.5%, 88.0%; 0.49	≤30.84 ng/mL; 59.6%, 85.0%; 0.45
Never smokers, *H. pylori* negative	≤1.81; 81.6%, 92.8%; 0.74	≤16.00 ng/mL; 73.5%, 94.5%; 0.68
Former smokers, *H. pylori* positive	≤2.21; 65.9, 83.5%; 0.49	≤33.51 ng/mL; 54.5%, 84.5%; 0.39
Former smokers, *H. pylori* negative	≤2.51; 92.6%, 92.4%; 0.85	≤22.75 ng/mL; 85.2%, 94.9%; 0.80
Current smokers, *H. pylori* positive	≤2.07; 56.0%, 85.2%; 0.41	≤40.91 ng/mL; 54.0%, 76.9%; 0.31
Current smokers, *H. pylori* negative	≤3.01; 48.4%, 86.7%; 0.35	≤37.75 ng/mL; 67.7%, 71.7%; 0.39

Pg—pepsinogen.

**Table 6:** Sensitivity and specificity of pre-existing and new pepsinogen cut-off values for current smokers by *H. pylori* presence.

Pg Cut-Off; Sensitivity (%), Specificity (%)		
	Pg I/Pg II	Pg I (ng/mL)
Current smokers, *H. pylori* positive, pre-existing cut-offs	PgI/II ≤ 2;	PgI ≤ 30 ng/mL;
	52.0 (37.4–66.3),	32.00 (19.52–46.70),
	87.0 (79.2–92.7); AUC 0.76	91.7 (84.8–96.1); AUC 0.63
New cut-offs	≤2.07; 56.0, 85.2; JJ 0.41	≤40.91 ng/mL; 54.0%, 76.9%; JJ 0.31
Current smokers, *H. pylori* negative, pre-existing cut-offs	PgI/II ≤ 2; 32.3 (16.7–51.4),	PgI ≤ 30 ng/mL; 38.7 (21.9–57.8),
	93.3 (83.8–98.2); AUC 0.68	86.7 (75.4–94.1); AUC 0.68
New cut-offs	≤3.01; 48.4, 86.7; JJ 0.35	≤37.75 ng/mL; 67.7%, 71.7%; JJ 0.39

Pg—pepsinogen, CI—95% confidence interval, AUC—area under ROC curve, JJ—Youden’s index.

## Data Availability

Publicly archived datasets are not currently available for the study.

## Supplementary Materials

The following supporting information can be downloaded at: https://www.mdpi.com/article/10.3390/diagnostics12051166/s1, Table S1: Multivariable regression model for false negative cases in detecting precancerous gastric lesions with pepsinogen testing (*n* = 293); Table S2: Comparison of median Pg I values by smoking status and *H. pylori* presence for participants with and without precancerous gastric lesions.
