# Supplementary material for: Factors Associated with False Negative Results in Serum Pepsinogen Testing for Precancerous Gastric Lesions in a European Population in the GISTAR Study
Source: Diagnostics (Basel). 2022 May 7;12(5):1166. doi: 10.3390/diagnostics12051166 (PMC9139962; doi:10.3390/diagnostics12051166)
Supplement: Supplementary file 1 [file diagnostics-12-01166-s001.zip › diagnostics-1684953-supplementary.pdf]

Supplementary Materials

Table S1. Multivariable regression model for false negative cases in detecting precancerous gastric lesions with pepsinogen testing (n=293)

Variables, n (%)	OR	95% CI	p – value
Sex (male)	0.94	0.51-1.74	0.85
Age (years)	1.01	0.97 – 1.05	0.77
Income (Euros)[a]<br><250<br>250-500<br>>500	 ref<br>0.58<br>0.57	 <br>0.33 – 1.01<br>0.24 – 1.39	 <br>0.05<br>0.22
Never smokers<br>Former smokers<br>Current smokers	Ref<br>1.07<br>3.22	 0.51-2.25<br>1.57-6.58	 0.86<br><0.01
Alcohol (per 10g increase/week)	1.07	1.01-1.14	0.03
At least 400g fruit and vegetables daily	1.03	0.60-1.75	0.93
Onion and spring onion			
once a week<br>2-4 times per week<br>5-6 times per week<br>every day	Ref<br>1.62<br>0.90<br>1.43	 0.72-3.64<br>0.38-2.14<br>0.57-3.58	 0.24<br>0.81<br>0.45
Coffee			
once a week<br>2-6 times per week<br>every day	Ref<br>0.65<br>0.77	 0.26-1.63<br>0.36-1.67	 0.36<br>0.51
BMI	0.99	0.70-1.39	0.93
H. pylori positive (biopsy)	2.54	1.50-4.28	<0.01
PPI use previous month	1.05	0.40-2.76	0.93

The false negative group (FN) was compared against the true positive (TP) group.
[a] monthly household income per household member after tax.
OR – odds ratio, adjusted for all the factors in the model, CI – confidence interval, IQR – interquartile range, PPI – proton pump inhibitors, ref – reference value.

Table S2. Comparison of median Pg I values by smoking status and *H. pylori* presence for participants with and without precancerous gastric lesions

	No precancerous lesions PgI ng/mL median, IQR			Precancerous lesions PgI ng/mL median, IQR		
	H. pylori positive	*H. pylori* negative	p value*	*H. pylori* positive	*H. pylori* negative	p value*
study population	51.51 IQR 26.7	37.87 IQR 19.31	<0.0001	30.28 IQR 34.69	8.59 IQR 20.63	<0.0001
never smokers	51.48 IQR 26.58	35.13 IQR 17.28	<0.0001	26.32 IQR 33.45	7.22 IQR 14.13	<0.0001
former smokers	53.14 IQR 27.55	40.41 IQR 17.04	<0.0001	30.11 IQR 38.92	8.7 IQR 8.88	<0.0001
current smokers	50.99 IQR 25.21	46.08 IQR 18.83	0.04	40.47 IQR 38.54	34.34 IQR 33.69	0.12

*Differences obtained using Mann-Whitney U test to compare *H. pylori* positive and negative groups. IQR – interquartile range.
